# Differential Deactivation during Mentalizing and Classification of Autism Based on Default Mode Network Connectivity

**DOI:** 10.1371/journal.pone.0050064

**Published:** 2012-11-19

**Authors:** Donna L. Murdaugh, Svetlana V. Shinkareva, Hrishikesh R. Deshpande, Jing Wang, Mark R. Pennick, Rajesh K. Kana

**Affiliations:** 1 Department of Psychology, University of Alabama at Birmingham, Birmingham, Alabama, United States of America; 2 Department of Psychology, University of South Carolina, Columbia, South Carolina, United States of America; Hangzhou Normal University, China

## Abstract

The default mode network (DMN) is a collection of brain areas found to be consistently deactivated during task performance. Previous neuroimaging studies of resting state have revealed reduced task-related deactivation of this network in autism. We investigated the DMN in 13 high-functioning adults with autism spectrum disorders (ASD) and 14 typically developing control participants during three fMRI studies (two language tasks and a Theory-of-Mind (ToM) task). Each study had separate blocks of fixation/resting baseline. The data from the task blocks and fixation blocks were collated to examine deactivation and functional connectivity. Deficits in the deactivation of the DMN in individuals with ASD were specific only to the ToM task, with no group differences in deactivation during the language tasks or a combined language and self-other discrimination task. During rest blocks following the ToM task, the ASD group showed less deactivation than the control group in a number of DMN regions, including medial prefrontal cortex (MPFC), anterior cingulate cortex, and posterior cingulate gyrus/precuneus. In addition, we found weaker functional connectivity of the MPFC in individuals with ASD compared to controls. Furthermore, we were able to reliably classify participants into ASD or typically developing control groups based on both the whole-brain and seed-based connectivity patterns with accuracy up to 96.3%. These findings indicate that deactivation and connectivity of the DMN were altered in individuals with ASD. In addition, these findings suggest that the deficits in DMN connectivity could be a neural signature that can be used for classifying an individual as belonging to the ASD group.

## Introduction

Neuroimaging studies have consistently shown altered patterns of functional brain activation and connectivity during cognitive tasks in individuals with autism spectrum disorders (ASD) (e.g., [1], [2], [3]). Of late, studies have begun to provide evidence that the brain in ASD is also functionally different during rest compared to typically developing individuals [4], [5], [6], [7], [8], [9]. In healthy, typically developing individuals, a distinct network of cortical midline structures are consistently deactivated when the individual is engaged in a cognitively demanding, goal-oriented task [10], [11]. These regions include the medial prefrontal cortex (MPFC), ventral anterior cingulate cortex (ACC), posterior cingulate cortex (PCC), precuneus (PrC), angular gyrus (AG), and bilateral inferior parietal lobules (IPL), among others, and are collectively referred to as the default mode network (DMN) [12], [13], [14], [15], [16], [11].

Interestingly, many of the DMN regions are the same regions that are found to be activated in studies of self-reference and ‘theory of mind’ (ToM) [17, 18, 19. 20, 21]. Specifically, the MPFC has been shown to play a distinct role in ascribing mental states to others [18], [19]. Indeed, the DMN has been hypothesized to be involved in internal mentalizations that help individuals navigate their social environment by attributing mental states (beliefs, desires, and thoughts) to oneself and to others, the ability to rehearse social narratives to engage in interactions with others, and in imagining the future [17], [16], [22]. As both behavioral and neuroimaging studies have successfully shown, processes like ToM and self-referential thinking are atypical in autism [23], [24], [1], [2], suggesting that such social deficits in autism may be pervasive even at rest.

Neuroimaging investigations of the ‘default mode’ brain function have collectively found altered recruitment and connectivity of the DMN in autism, and support the hypothesis that it may partially explain deficits in social interactions that are characteristic of individuals with ASD. Two common techniques used in studying the DMN are: task-induced deactivation, and continuous resting state scan. Task-induced deactivation involves analyzing data from resting blocks in an fMRI study by comparing them to the interleaving task blocks [4], [6], [7]. These studies have found that individuals with ASD have lower levels of functional deactivation of the DMN than typical control participants [7], especially in the MPFC and ACC regions that appear to be independent of task performance [6]. Functional connectivity results have suggested that the deficits of the DMN in ASD are mainly between relatively long-range anterior to posterior connections [4]. The other technique involves analysis of data from a continuous resting state scan [5], [8], [9] and has found similar results to that of task-induced deactivation.

The task-induced deactivation technique provides a unique venue to examine the effect of a cognitive task on the DMN. Indeed, Gusnard et al. [25] suggested that there is a differential deactivation of the DMN when a cognitive task involves more self-referential thought processes, such that the dorsal MPFC is activated, while the ventral MPFC is deactivated. Even though deactivation of the DMN is considered to be non-specific to task, many self-referential tasks will evoke activation of regions similar to the DMN [16]. As such, tasks that target social cognition and ToM may show a different DMN pattern than other cognitive tasks because of these shared brain regions [15]. Kennedy and Courchesne [6] suggested that there may be both task-specific and task-independent deficits of the DMN in people with ASD. In particular, they found that task-specific deficits in DMN deactivation in ASD only occurred in the task that required the participants to make judgments based on either internal personality traits or external observable traits. Indeed, assessing deactivation of the DMN during a ToM-based task may help simulate a real-world environment in which deficits in DMN may contribute to dysfunctions in social interactions that would occur in real life. In addition, it may be that altered deactivation of the DMN in people with ASD only occurs during tasks in which ASD individuals already show marked impairments, such as ToM tasks.

In the present study, we used task-induced deactivation combined from multiple functional magnetic resonance imaging (fMRI) studies in order to assess the alterations of the DMN in individuals with ASD and the effect of the preceding task on the DMN. We assessed three types of tasks, language comprehension, self-reference with a language component, and ToM (without a language component), all of which are shown to evoke atypical cognitive and brain responses in individuals with ASD (e.g., [26], [1]). The main goal was to determine whether deficits in the DMN in ASD could be seen during deactivation of multiple tasks in which individuals with ASD have particular difficulty, or whether the deficits in the DMN were limited only to the ToM task, which relies more on self-other reference. We hypothesized that, similar to previous findings [7], individuals with ASD would have overall lower levels of deactivation of the DMN than typical control participants. Secondly, we hypothesized that when the tasks were divided to assess deactivation of the DMN separately for ToM versus language comprehension tasks, individuals with ASD would only show a deficit in the task that required processing ToM compared to controls. As suggested above, we hypothesized that the DMN deficits would be more pronounced during ToM because it involves mentalizing and self-other referential thinking, which is thought to be mediated by the DMN [17], [16], [22,], and has been shown to be difficult for individuals with ASD (e.g., [24], [23], [27]. As such, we were also interested in how much of a role language plays in self-reference and hypothesized that a language task that also contains a self-other discrimination component will produce a deficit in deactivation in individuals with ASD compared to controls, but not to the extent of the ToM task that has no language component.

We were also keen to further examine whether altered functional connectivity seen in individuals with ASD could successfully distinguish between a DMN of a typically developing individual from that of an individual with ASD. Kennedy and Courchesne [5] found that abnormalities in functional connectivity of the DMN were related specifically to weaker connectivity of the MPFC in individuals with ASD. Recently, Assaf et al. [9] also found decreased functional connectivity between the MPFC/ACC and more posterior regions, such as the PrC, and these decreases were correlated with greater severity of social symptoms in people with ASD. Similar findings were also reported in Monk et al. [8], where decreased connectivity between the PCC and SFG was correlated with greater social dysfunction in individuals with ASD. With these converging results, it stands to reason that alterations in functional connectivity of the DMN could be used as an index to successfully classify individuals as being on the autism spectrum or not. We hypothesized (1) that weaker functional connectivity would be seen in relatively more anterior brain regions, such as the MPFC, thus contributing to existing findings, specifically Kennedy and Courchesne [5], and (2) that the differences between the DMN pattern of functional connectivity in ASD individuals and controls would be sufficient to successfully classify an individual into the ASD or control group.

## Methods

### Ethics Statement

The research reported in this manuscript has been approved by the Institutional Review Board of the University of Alabama at Birmingham. The IRB review was conducted in accordance with UAB’s Assurance of Compliance approved by the Department of Health and Human Services. Signed written informed consent (approved by the institutional review board) was obtained from all participants who took part in this study.

### Participants

Thirteen high-functioning young adults with ASD (all male, one left-handed) and fourteen typical control participants (all male, right-handed) were included in this study. Participants, both ASD and controls, were recruited from the local community and from the University of Alabama at Birmingham (UAB) using flyers and an advertisement in the UAB Reporter newspaper classifieds. Many of the participants with ASD were recruited through the UAB Civitan-Sparks Clinic and through the University of Alabama Autism Spectrum Disorders Clinic database and surrounding service providers in the state of Alabama. They had received a diagnosis of an ASD based on the Autism Diagnostic Interview-Revised (ADI-R) [28] symptoms, Autism Diagnostic Observation Schedule (ADOS) [29], and clinical impressions. Six of the 13 participants with ASD in this study had received a diagnosis of Asperger’s Disorder. The ASD and control participants did not significantly differ in age (means±SD, ASD: 21.4±3.9 and control: 22.6±4.2, *t*(25) = −.728, *p* = .473). The mean Wechsler Abbreviated Scale of Intelligence (WASI) full scale intelligence quotients for the two groups were not significantly different (ASD: 105.2±17.7 and control: 113.3±8.4, *t*(25) = −1.336, *p* = .194). Three of the 13 participants with ASD were being treated with medication when scanned: one received a selective norepinephrine reuptake inhibitor, one a CNS stimulant, and one an antipsychotic. Participants were excluded on the basis of metal implanted in their bodies (either surgically or accidentally), history of kidney disease, seizure disorder, diabetes, hypertension, anemia, or sickle cell disease, or being claustrophobic. All participants or their legal guardians gave written informed consent, approved by the UAB Institutional Review Board, to participate in the study and were compensated for their participation.

### Experimental Stimuli

The default mode network was assessed by combining data from three separate fMRI studies in ASD. Two of the studies consisted of stimuli that assessed word and sentence comprehension, and the third study was designed to assess ‘theory of mind’ (ToM) without a language component. In the sentence comprehension study, the participants were presented with sentences that contained a pun word or a word with literal meaning. The participants were instructed to press a button after they finished reading each sentence. All sentences were matched in terms of length (number of words) and were presented in a blocked design format (four blocks of pun and four blocks of literal sentences). Prior to each block of puns, participants were shown a cue stating “two meanings” and for each block of literal sentences, a cue stating “one meaning” prompting the participants about the task. Each sentence was presented for 5000 ms with an inter-trial interval of 1000 ms and each block consisted of six sentences. The blocks were presented in a pseudo-randomized order during the course of one scanning session. This experiment also included five fixation blocks, each lasting 24 seconds.

In the word comprehension study, participants were presented with a series of words (e.g., honest, intelligent, smart, happy) and were instructed to determine whether the words described themselves (in some blocks) or the words described their favorite teacher (in other blocks). In the control condition, participants were instructed to determine whether a word contained a specific letter (case judgment control condition). The experiment was presented in a blocked design format with the order of presentation of blocks counterbalanced across participants. Stimuli were positive and negative adjectives. The three judgment tasks were completed in a blocked design format with three blocks for each type of judgment. There were seven fixation blocks in this experiment, each lasting 15 seconds. This experiment had both a language component and a self-other discrimination component, which has been shown to activate parts of the default mode network [23], [24], [1], [2].

In the third study, designed to assess the ToM network, the participants were presented with a series of black and white comic strip vignettes (adapted from [30]) depicting scenarios that demanded either a physical causal attribution or an intentional causal attribution. The first part of the vignette was presented for 5 seconds and the participants’ task was to choose a logical ending to the story from the three choices in the second panel presented for 6 seconds. The whole vignette remained on the screen for a total of 11 seconds. Participants were to indicate the answer (A, B, or C) by a button press. Participants viewed a total of 11 physical cartoons, and 11 intentional cartoons. There were five fixation epochs in this experiment, each lasting 24 seconds.

Interlaced among the two to three cognitive tasks of each study were a total of 17 rest (fixation) periods, each presented for 15 or 24 seconds, in which the participants were asked to relax, and passively view an asterisk presented on the middle of the screen. Participants performed all three studies during the same scanning session. The data from these separate fMRI studies (task blocks and rest blocks) were collated to examine brain deactivation and functional connectivity using previously established methods of task-induced deactivation [4], [31].

### Data Acquisition

Functional and structural MRI data were collected at the UAB Civitan International Research Center on a Siemens 3.0 Tesla Allegra head-only scanner (Siemens Medical Inc., Erlangen, Germany). For structural imaging, initial high resolution T1-weighted scans were acquired using a 160-slice 3D MPRAGE volume scan with TR = 200 ms, TE = 3.34 ms, flip angle = 12 degrees, FOV = 25.6 cm, 256×256 matrix size, and 1 mm slice thickness. For functional imaging, a single-shot gradient-recalled echo-planar pulse sequence was used for rapid image acquisition (TR = 1000 ms, TE = 30 ms, flip angle = 60 degrees). Seventeen adjacent oblique-axial slices were acquired in an interleaved sequence with 5 mm slice thickness, 1 mm slice gap, a 24×24 cm field of view (FOV), and a 64×64 matrix, resulting in an in-plane resolution of 3.75×3.75×5 mm. The stimuli were rear-projected onto a translucent plastic screen and participants viewed the screen through a mirror attached to the head coil.

### fMRI Data Analyses: Distribution of Deactivation

For each of the three studies, data were pre- and post-processed and statistically analyzed using SPM8 (Wellcome Department of Cognitive Neurology, University College London, London, UK). Functional images were slice time-corrected to the onset of the middle slice and spatially realigned. After realignment, a mean functional image was computed for each separate study and then matched to the EPI template provided within SPM8. Data were then spatially normalized to standard Montreal Neurological Institute (MNI) brain space and spatially smoothed using a three-dimensional Gaussian kernel of 8 mm full-width at half-maximum (FWHM). Statistical analyses were performed on individual and group data using the general linear model, while group analyses were performed using a random-effects model. Clusters with statistically significant deactivation were identified using a *t*-statistic on a voxel by voxel basis. Separate regressors were created for the combined language conditions, the ToM condition, and the rest (fixation) by convolving a boxcar function with the standard hemodynamic response function as specified in SPM8. For each individual subject, contrasts were generated separately for each set of rest blocks specific to the task it is being contrasted with, *fixation (*henceforth rest*) vs. language tasks* (which included pun sentences, literal sentences, and word-letter identification, collectively referred to from this point on as language tasks), *rest vs. self-other discrimination language task* (which included both a language (word) component and a self-other discrimination component), and *rest vs. ToM task* (which contained no language component). In addition, contrasts were generated for each subject using the IMCALC function in SPM8 for *rest vs. all cognitive tasks*. Statistical maps were superimposed on normalized T1-weighted images.

To determine significant differences between the two groups, a regions of interest (ROI) approach was used. These ROIs were defined functionally to encompass the main clusters of deactivation based on the combined ASD and control groups’ deactivation of the default mode network, and was done in order to improve statistical power and address our *a priori* hypotheses. These functional ROIs included: posterior cingulate cortex/precuneus (PCC/PrC), anterior cingulate cortex (ACC), medial prefrontal cortex (MPFC), dorsolateral prefrontal cortex (DLPFC), superior temporal gyrus (STG), and angular gyrus (AG). For cluster level inference, data were intensity-thresholded such that the false discovery rate (FDR) was ≤0.05 for number of contiguous voxels with p<0.001 or p<0.005. For direct comparison between the ASD and control groups, using our ROI approach, we used p<0.001 with a cluster threshold of 110 contiguous voxels.

### fMRI Data Analysis: Functional Connectivity and Classification of Participants

The functional connectivity (synchronization of the time course of activation across brain areas) analyses were conducted using SPM8 and the REST toolbox [32]. In order to facilitate the analysis of “resting state” in a blocked-design study in the same manner as continuous resting state data, we removed the interleaving task blocks as described by Fair et al. [31]. The first 6 sec of each rest block was removed in order to account for the hemodynamic response to return to baseline. Also, the first 6 sec of the task block was included in the study that had the 15 sec rest blocks in order to account for the hemodynamic delay. The resting state data were then collated for each individual subject. The motion corrected fMRI data were normalized to the standard MNI template and resampled to an isotropic 3 mm^3^ voxel. In addition, the mean head motion parameter for all participants were determined and included in the analyses as a separate regressor to account for the variance related to head movement. Data were also processed to reduce additional variance that may not reflect neuronal activity. The linear trend and mean within each session was removed. The mean signal in gray matter, white matter, cerebrospinal fluid and the whole brain were partialled out along with the six rigid body head movement parameters as regressors of no interest [31]. The gray matter, white matter and cerebrospinal fluid were defined by anatomical masks using WFU PickAtlas [33], [34].

Three task-induced deactivation seed regions were defined using the same coordinates as those found by both Fox et al. [14] and Kennedy and Courchesne [5], and were previously identified as the three most significant regions in a meta-analysis of task-induced deactivation [12]. These three seed regions are: the medial prefrontal cortex (MPFC; −1, 47, −4), posterior cingulate/precuneus (PCC/PrC; −5, −49, 40), and the left angular gyrus (AG; −45, −67, 36). Functional connectivity was computed (separately for each participant) by correlating the average time course of signal intensity of each seed region with all other voxels in the brain. One-sample t-tests were then conducted for each of the seed regions separately for both the control the ASD groups in order to find significant correlations with the seed region and other brain regions. Each of the three seed region correlation coefficients were converted to a normal distribution using the Fisher’s *r* to *z* transformation. The three seed regions were then averaged separately for the ASD and control group to obtain a DMN connectivity map for each group [5]. A two-sample t-test of the *z* scores within SPM8 was conducted to assess group differences between the control and ASD groups’ DMN connectivity maps. All data were intensity-thresholded at *z* = 3.53, *p*<0.001, with a cluster size correction per region for a voxel-wise family wise error (FWE) rate of 0.05. To determine the voxel threshold for significance, a minimum cluster thresholding operation was performed using the *AlphaSim* software package in AFNI (Analysis of Functional Neuroimages) [35]. Ten-thousand Monte Carlo simulations were generated to maintain the family wise error (FWE) rate at 0.05 for the whole brain.

The classification analysis was conducted using in-house scripts in Matlab (2010, The MathWorks, Natick, MA). The purpose was to examine whether the resting state functional connectivity could indicate a participant’s group membership as ASD or control, based on the connectivity patterns of the others. We performed classifications on two types of functional connectivity: the connectivity between *a priori* seed regions and anatomically defined ROIs (referred to as seed-based connectivity below), and the pairwise connectivity between anatomically defined ROIs (referred to as full connectivity matrix below). The three seed regions, AG, MPFC, and PCC, were the same as described above. For the seed-based connectivity, voxels included in the seeds were removed from the corresponding ROIs prior to connectivity computation. The anatomical ROIs were defined by the automated anatomical labeling (AAL) [36]. Among the 116 regions in the template, 14 regions, mostly in the vermis and cerebellum, were excluded due to missing functional data, resulting in 102 ROIs in total.

To determine if a participant can be identified as autism or control based solely on the resting state connectivity of others, the participants were assigned into training and test sets, with each participant treated as the test set iteratively. Logistic regression classifier was trained on all but one participant’s connectivity maps without further feature extraction, and tested on the left-out participant to identify the individual as autism or control [37]. The significance of classification accuracy was evaluated based on the binomial distribution B(n, p), where n is the number of participants and p is the probability of correct classification when the participants are randomly labeled [37].

## Results

### Overview

The key findings of this study are: 1) Within-subject analyses revealed that control participants exhibited stronger deactivation in key regions of the DMN (ventromedial prefrontal, anterior cingulate, posterior cingulate cortices) during task-related performance. Participants with ASD, however, showed DMN deactivation limited to the posterior cingulate cortex and precuneus; 2) Between-group analyses revealed that participants with ASD showed lower levels of deactivation in DMN areas (e.g., left medial prefrontal cortex) during rest; 3) When the tasks were separated to examine rest blocks following social cognitive or linguistic tasks, using a Group (ASD, Control)×Tasks (Fixation, Language, Self-referential language, ToM) ANOVA, rest blocks following the language tasks and self-other discrimination language task revealed no group difference. However, rest blocks that are part of the ToM task showed less deactivation in ASD participants than in controls in several DMN regions (e.g., left superior frontal gyrus, posterior cingulate). 4) Functional connectivity results revealed significantly reduced connectivity of the MPFC in ASD participants than controls. 5) Pattern classification results revealed 77.78% accuracy in identifying autism or control individuals based on patterns of functional connectivity.

### Distribution of Deactivation

For the *rest vs. all cognitive tasks* contrast, the whole-brain analysis for the control participants revealed significant regions of deactivation in classic default mode network areas, including the LACC, bilateral PCC/PrC, MPFC, and left angular gyrus (p<0.001, FDR≤0.05) (Table 1). However, similar to previous findings in autism (e.g. [7], [6]), greater deactivation in the rest condition compared to the cognitive tasks in participants with ASD was limited to relatively posterior regions, specifically the PCC/PrC. For a summary of brain regions activated for each task, please refer to Supplemental Material (Figure S1 and S2).

**Table 1:** Within-group deactivation during all cognitive tasks combined for the ASD (n = 13) and control (n = 14) participants (p<0.001 uncorrected, FDR cluster-corrected).

Group	Brain Region	BA^a^	Hem^b^	*k* c	x^d^	y	z	t	*p*, FDR^e^
Control	Posterior Cingulate Cortex	31	R	6409^f^	8	−42	40	13.10	3.45×10^−22^
	Precuneus		L	6409^f^	−8	−40	46	11.84	3.45×10^−22^
	Anterior Cingulate Cortex	32	L	2742^g^	−4	40	8	11.81	1.02×10^−12^
	Medial Prefrontal Cortex	10	L	2742^g^	−6	50	−2	11.17	1.02×10^−12^
	Dorsolateral Prefrontal Cortex	10	R	791	30	36	44	11.02	1.16×10^−5^
		9	L	368	−24	38	42	6.71	0.002
	Angular Gyrus		R	208	48	−70	34	6.22	0.012
	Superior Temporal Gyrus		R	249	66	−26	8	5.07	0.007
**Group**	**Brain Regions**	**BA**	**Hem**	***k***	**x**	**y**	**z**	**t**	***p*** **, FDR**
ASD	Precuneus	31	L	140	−12	−60	28	6.68	0.036
	Posterior Cingulate Cortex		R	216	12	−56	26	6.63	0.012
			R	325	12	−38	40	3.67	0.004

a Brodmann area.

b Hemisphere: R, right, L, left.

c Number of contiguous voxels with p<0.001.

d x, y, and z coordinates in MNI space.

e False discovery rate corrected at the individual cluster level.

f,g Regions of activation encompassed within the same cluster.

**Figure 1:**
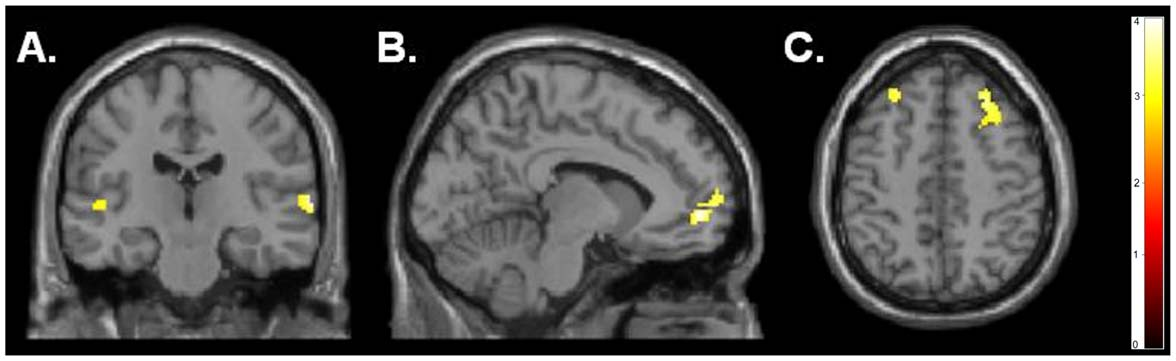
Regions of Interest showing greater deactivation for control participants compared to participants with ASD for the contrast of rest blocks>all tasks combined (language, self-referential langauge, and ToM) in the (A) bilateral STG (68, −22, 4; −52, −62, 26), (B) LMPFC (−2, 66, 10), and (C) bilateral DLPFC (−42, 40, 18; 28, 30, 48) (p<0.005, cluster-corrected at 110 voxels).

Direct comparison of the two groups using our ROI approach revealed overall significantly lower deactivation during rest periods in participants with ASD than in typical control participants. The ROI analysis revealed significantly less deactivation in the participants with ASD when compared to controls in DMN regions, specifically, the bilateral superior temporal gyrus (RSTG), LMPFC, and bilateral DLPFC (p<0.005, cluster-corrected at 110 voxels) (Figure 1). No significantly greater activity was seen in the ASD group when compared to the control group.

**Figure 2:**
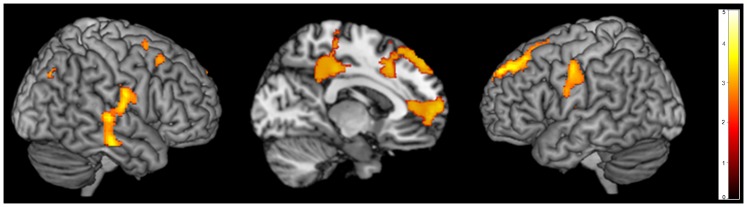
Brain areas showing greater deactivation for control participants compared to participants with ASD for the contrast of rest>ToM task in the LMPFC, LACC, LSFG, LSTG, DLPFC, and PCC/PrC (p<0.001 uncorrected, FDR cluster-corrected).

In order to determine the influence of the preceding task on the resting blocks in the two groups, the cognitive tasks were further entered into a Group (ASD, control)×Condition (rest vs. language, rest vs. self-other language, rest vs. ToM) ANOVA using the full factorial model in SPM8. This analysis indicated a main effect of group with participants with ASD showing overall decreased deactivation compared to controls (FWE≤0.05). A main effect of condition revealed that the rest periods preceded by a language task, including the self-other discrimination language task, exhibited greater overall whole-brain deactivation than rest periods preceded by the ToM task (FWE≤0.05). In order to further examine task deactivation, we conducted tests of the simple main effects of group within each condition, such that we compared each task to their own set of rest blocks, each in a separate single analysis, assessing each task’s deactivation separately for between group differences. These results demonstrated that during rest blocks following the ToM task, for the contrast fixation (rest) vs. ToM, the ASD group showed less deactivation than the control group in a number of DMN regions, including LMPFC, ACC, LSFG, DLPFC and PCC/PrC (Figure 2; Table 2) (p<0.005, FDR ≤0.05). However, interestingly, there were no group differences in the rest blocks preceded by the language tasks or the rest blocks preceded by the self-other discrimination language task. A few possible interpretations for these findings are explored in the discussion.

**Table 2:** Brain areas showing greater deactivation for control participants (n = 14) compared to participants with ASD (n = 13) for the contrast of rest>ToM task (p<0.005 uncorrected, FDR cluster-corrected).

Brain Region	BA^a^	Hem^b^	*k* c	x^d^	y	z	t^e^	t_ASD_ f	t_CON_ g	*p*, FDR^h^
Superior Frontal Gyrus	8	L	1380^i^	−20	46	44	4.10	−1.66	4.78	0.004
Dorsolateral Prefrontal Cortex	9	L	1380^i^	−14	56	38	4.18	−2.66	3.38	0.004
Medial Prefrontal Gyrus		L	870^j^	−6	64	10	4.50	−0.68	7.38	0.015
Anterior Cingulate Cortex	10	L	870^j^	−10	56	2	4.63	0.02	6.98	0.015
		L	870^j^	−14	46	−6	3.88	0.03	8.28	0.015
Middle Temporal Gyrus	21	R	1105	64	−20	−18	4.10	−2.84	4.06	0.007
Posterior Cingulate Cortex		L	611^k^	−8	−32	38	4.06	−1.86	4.17	0.039
	31	R	611^k^	2	−44	34	3.46	1.65	6.16	0.039
Precuneus	7	L	611^k^	−6	−36	48	4.06	−1.64	3.67	0.039

a Brodmann area.

b Hemisphere: R, right, L, left.

c Number of contiguous voxels with p<0.005.

d x, y, and z coordinates in MNI space.

e peak t value for the between group analysis.

f t vaule for the within-group analysis for the ASD group at the peak coordinates represented in the between-group analysis.

g t vaule for the within-group analysis for the control group at the peak coordinates represented in the between-group analysis.

h False discovery rate corrected at the individual cluster level.

i,j,k Regions of activation encompassed within the same cluster.

### Functional Connectivity Results

**Figure 3:**
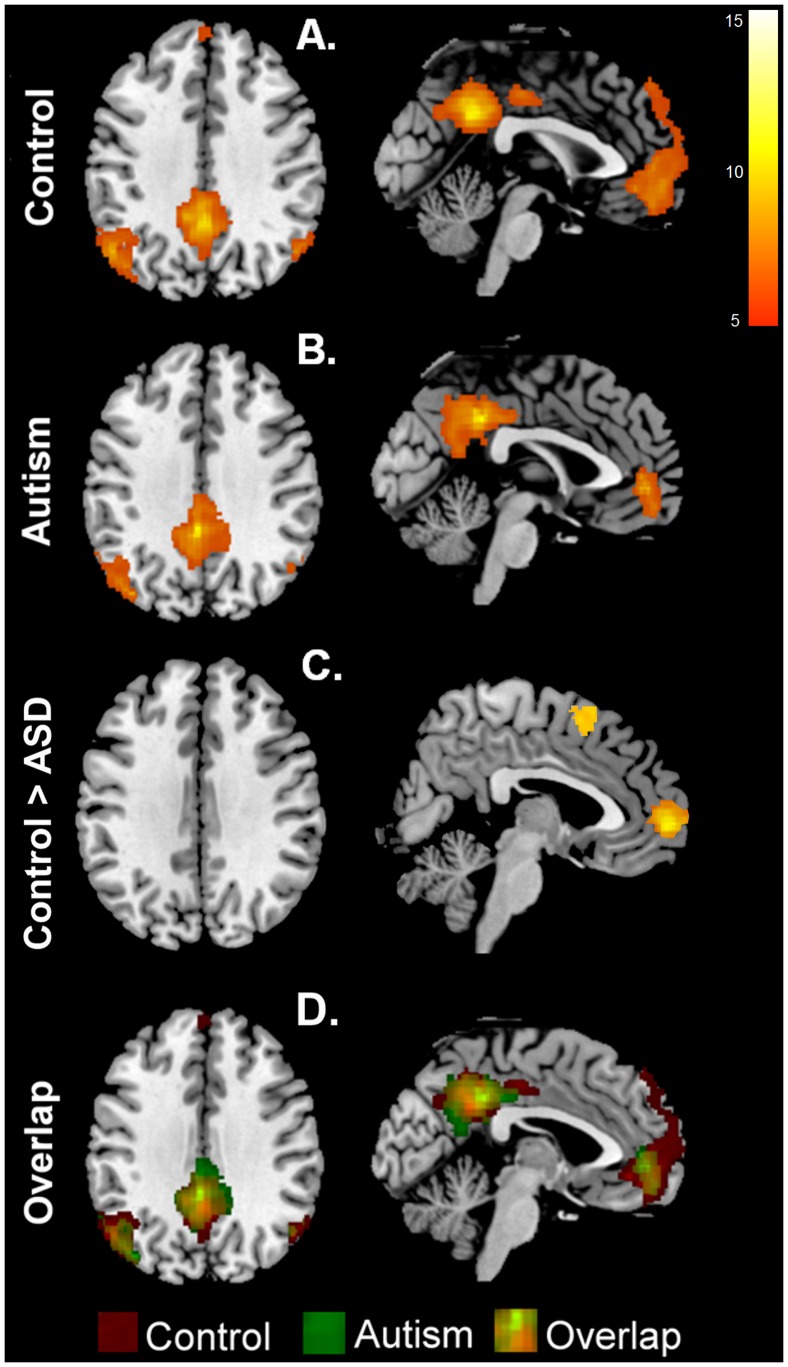
The averaged DMN connectivity map (p<0.001, cluster corrected). Significant clusters shown are represented as z scores. (A) DMN map for the control group. (B) DMN map for the ASD group. (C) DMN map of regions with greater connectivity in control participatns than participants with ASD. (D) Overlap of the control and ASD connectivity maps.

Our functional connectivity results revealed a similar DMN connectivity map for our control group as reported by Fox et al. [14], Kennedy and Courchesne [5], and Assaf et al. [9]. The DMN connectivity map for the control participants included the bilateral PCC/PrC, including the adjacent region of the postcentral gyrus, bilateral AG, left MPFC, extending into SFG, and bilateral STG (Figure 3a; *p*<0.001, cluster-corrected at 32 voxels). For the ASD group, the DMN connectivity map included the left PCC/PrC, bilateral MPFC, and right IPL (Figure 3b; p<0.001, cluster-corrected at 32 voxels). Indeed these results suggest that the control group has an extensive and robust connectivity of the DMN similar to other studies that have assessed the DMN network in healthy adults (e.g., [9], [11]). The ASD group on the other hand showed connectivity that is more limited and different from that seen in typically developing individuals. Our between-group results revealed that the ASD group had significantly reduced DMN connectivity specific to the dorsal MPFC when compared to the control group (p<0.001, cluster-corrected at 32 voxels).

### Functional Connectivity: Pattern Classification Results

The classification analysis used functional connectivity as a benchmark for identifying unique patterns of connectivity for individual subjects as well as across groups. Participants’ group membership was identified with reliably accurate base on both types of connectivity.

#### Seed-based connectivity

**Figure 4:**
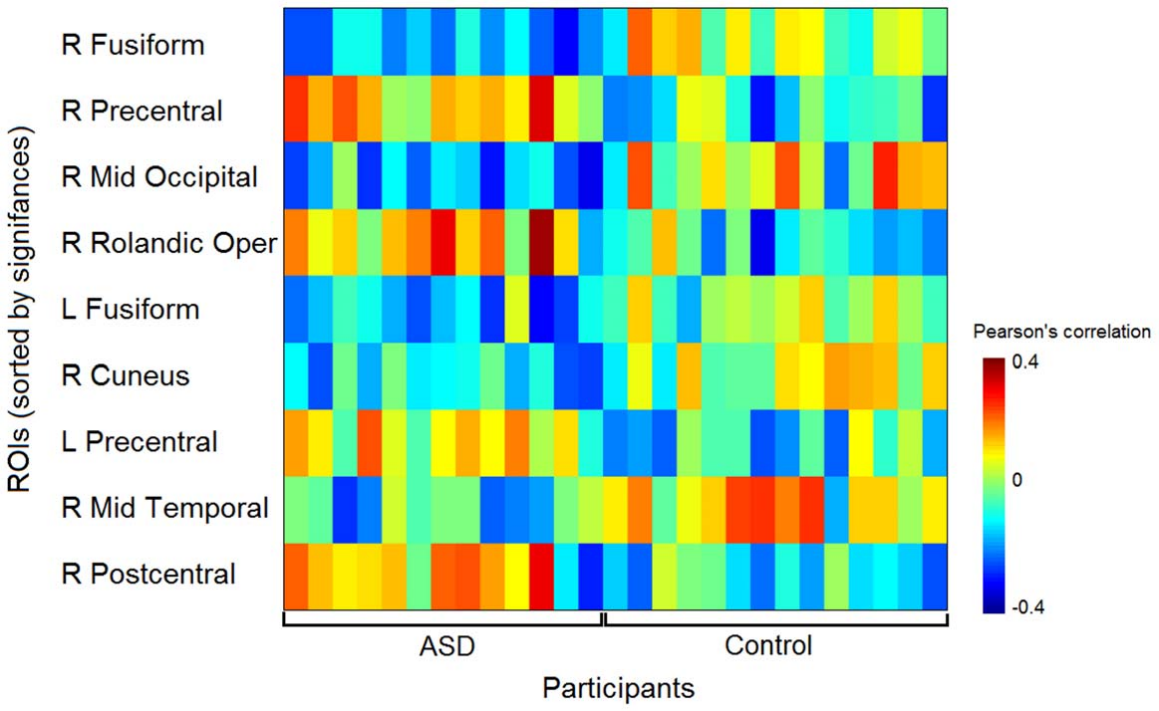
Correlations of resting-state time courses in which classification was based between ROI and seed region in the left AG, that showed significant group difference (*p* = 0.05, with Bonferroni correction), for each of the participants.

Classifications on AG-, MPFC-, and PCC- based connectivity maps resulted in accuracies of 96.30% (*p* = 0.000), 70.37% (*p* = 0.024), and 70.37% (*p* = 0.024) respectively. The connections with left AG and 9 ROIs showed significant differences between ASD and control group, based on two-sample t-tests, at *p* = 0.05 with Bonferroni correction (see Figure 4). The ASD group had significantly decreased functional connectivity compared to controls between the AG and visual regions, such as the right middle occipital lobe, right cuneus, as well as the right MTG, including bilateral fusiform gyrus. In contrast, the ASD group had significantly increased functional connectivity compared to controls between the AG and the bilateral precentral gyrus, right postcentral gyrus, and right rolandic operculum.

#### Full connectivity matrix

We were able to identify participants as belonging to ASD or control groups with 77.78% accuracy (*p* = .0006). Ten out of thirteen participants in the ASD group and eleven out of fourteen participants in the control group were correctly identified through this method (sensitivity: 76.9% and specificity: 78.6%). To investigate which connections contributed most to the participant identification, we examined the 0.5% of connections that contributed most to identification of ASD group and the top 0.5% of connections that contributed most to identification of control group, henceforth, the most informative connections. The left angular gyrus appeared in 10 out of the 66 most informative connections and was important for identification of ASD group. The right frontal operculum appeared in 7 out of the 75 most informative connections and was important for identification of control group (See Figure S3, S4 and Table S1 for a full list of the most informative connections). However, we note that classification accuracy was based on all of the connections, and caution should be taken in interpreting the connections with the highest classification weights [38].

## Discussion

The findings of this study indicate that individuals with ASD have both atypical deactivation of the DMN as well as underconnectivity of this network, specifically subserved by the MPFC. The deactivation of the DMN network was limited to relatively posterior brain regions in the ASD group, specifically the PCC/PrC. The between group analysis further confirmed this finding, with significantly less deactivation in the ASD group compared to the control group in primarily anterior brain regions, e.g., MPFC and DLPFC. In addition, the deficits in the deactivation of the DMN were specific only to the ToM task, with no group differences in deactivation during language comprehension or self-other discrimination. The functional connectivity analyses revealed three main findings. First, altered functional connectivity of the MPFC was found in ASD individuals compared to controls using a task-induced deactivation technique, thus replicating Kennedy and Courchesne [5] findings. This suggests that both the task-induced deactivation and continuous resting-state scanning technique are both adequate in detecting DMN differences in connectivity of ASD individuals versus a control group with adequate correction for interleaving task blocks [31]. Second, we were able to accurately classify individuals into ASD or control groups based on their differential DMN patterns of functional connectivity. Thirdly, classification based on functional connectivity was highest for the angular gyrus seed region with other regions of the brain.

The focus of the DMN activity in ASD in this study to the PCC/PrC is consistent with the findings of a few previous studies of functional activation of the resting state in ASD [7], [6]. In addition, the lack of deactivation of relatively anterior brain regions of the DMN found in ASD individuals is noteworthy considering the prefrontal cortex’s involvement in self-reflective thought, social and emotional judgments, and in the ability to attribute mental states to others [25], [39], [18], [19], and with the difficulty individuals with ASD have in comprehending such functions [24], [40], [23], [27]. However, it should be noted that Cherkassky et al. [4] did not report any differences between the groups in task-induced deactivation of the DMN despite finding connectivity differences. Their lack of deactivation differences may be due to the several different types of tasks, e.g., executive function, language, ToM, inhibition, etc., that were combined to assess resting state. Indeed, when we separated out type of task to assess influence of task on DMN deactivation, we found no differences between the ASD and control group in deactivation of the language comprehension tasks or the self-referential language task.

On the other hand, when we investigated deactivation of the DMN during the ToM task, which contained no language component, individuals with ASD had significantly less deactivation than controls in brain regions such as the LMPFC, ACC, LSFG, DLPFC and PCC/PrC. It is possible that reduced DMN deactivation during the ToM task reflects a pervasive deficit in the ability of individuals with ASD to engage in differential deactivation of the DMN when asked to attribute mental states to others. Indeed, it may be that the individuals with ASD did not engage in the task at all, or interpreted the task very differently than controls, resulting in altered patterns of deactivation from the control participants. This is plausible based on behavioral accuracy on this task; specifically, there were no differences between the ASD and control participants in percent accuracy (72.7% and 79.8% respectively). This may suggest that individuals with ASD may be able to accurately complete the task using deductive reasoning skills, while the control participants are more likely to interpret the task using social inferences. On the other hand, the similarity of DMN deactivation during the language tasks may simply reflect the fact that the verbal IQs between the ASD and control groups were not significantly different, suggesting that our high-functioning ASD individuals were perhaps able to engage cognitively in the task and deactivate the default system in a similar way as the controls. This idea is supported by the evidence provided above that introspection is difficult for individuals with ASD [24], [40], [23], [27].

A second plausible explanation for the group differences in the deactivation during the ToM task is that the control participants may just be more efficient in inferring the mental states of others. Indeed, the brain regions primarily implicated in ToM processing are the temporoparietal junction (TPJ), the temporal poles, the MPFC, and the PCC [41], [42], [18], [43]. In particular, Amodio and Frith [18] suggest that ToM processes occur specifically in the anterior rostral portion of the medial prefrontal cortex. In addition, Gusnard et al. [25] suggest that there is activation of the dorsal MPFC to self-referential mental activity, and subsequent attenuation of the ventral MPFC. These results suggest that healthy individuals are able to efficiently utilize the specific brain regions necessary for completing the ToM task and are able to modulate the deactivation of the DMN appropriately. The ASD individuals, on the other hand, may be unable to engage in mentalizing needed to infer the mental states of others, and as such are unable to modulate the recruitment of the DMN in regions specifically shared by both the ToM task and the default system, such as the MPFC, ACC, and PCC/PrC. The difficulty individuals with ASD may have in modulating DMN regions between ToM and resting scenarios may be related to cognitive difficulties they have. For instance, Frith and de Vignemont [44] suggest that self-related and other-related thinking may be mediated by egocentric and allocentric stances respectively and people with ASD may be affected by a disconnection between these two stances.

However, what is more interesting is that there were no differences between the ASD and control group for the combined task that included both a self-other discrimination component and a language component, which has also been shown to activate the DMN [45], [18]. A possible explanation for these results may be that individuals with ASD have intact DMN deactivation when asked to discriminate between self and others in this particular task. This explanation may be supported in part by Uddin’s [46] model of self in autism. According to this view, individuals with ASD have intact ability in processing certain aspects of self (e.g., “physical” attributes), such as self-recognition, but the breakdown in self-refection and mentalizing in ASD occurs in tasks that require “psychological” aspects of self, such as self-evaluation. For example, while individuals with ASD exhibit little to no differences from controls in such tasks as identifying self- versus other-faces [47], they struggle with self-other evaluations that involve personality traits or emotions [48]. It may be that in our self-other differentiation task, the participants may have focused on physical attributes more so than personality/psychological attributes, which would have been more difficult. As such, the individuals with ASD showed similar deactivation of the DMN as the controls, although the mechanism of how the descriptor words were mentalized may be quite different. When we combined all of the tasks that contained a language component, including the self-other task, our results were identical to if the self-other task was removed, with no group differences in deactivation of the DMN during these tasks. Nevertheless, our study did show that when a mentalizing task was presented without a language component, individuals with ASD had aberrant DMN deactivation compared to the controls. It may be that the language component of the self-other discrimination task masked the deficit of the DMN deactivation in individuals with ASD. Further research into the decomposition of the self in autism would be especially helpful in determining which functions seem to be intact and differentially deactivate the DMN and which ones are more difficult for individuals to modulate the recruitment of the DMN in regions specifically shared by both the self-referential task and the default system.

The functional connectivity results of the present study supplement the deactivation findings. The between group results revealed an overall decrease in functional connectivity of the MPFC in the ASD group compared to the control group. Specifically, the MPFC was the only region in the default mode connectivity network that showed significantly weaker connections between the ASD and control groups. Although the overall connectivity map of the ASD group only consisted of three brain regions, left PCC/PrC, bilateral MPFC, and right IPL, the only significant difference between the ASD and control group was in the dorsal MPFC. Interestingly, we used the same coordinates and default network averaging procedure as Kennedy and Courchesne [5] did for what they termed their “task negative network” (TNN). Our results replicate that of Kennedy and Courchesne [5] with many of the same DMN regions as their TNN regions with our task-induced deactivation technique (see [31]). Overall, our result again supports the idea that the deficit in connectivity of the dorsal MPFC in the ASD group may reflect a limited ability to self-reflect and mentalize [25], [18], [46]. Indeed, from a connectionist viewpoint, these results may partially explain our deactivation results. With lack of connectivity between the MPFC and more posterior regions during the default mode (as shown in previous studies, e.g., [4], [9]), individuals with ASD may have difficulty integrating the brain regions necessary for taking the mental perspective of another person, so that during tasks that require ToM, ASD individuals fail to modulate the deactivation of the DMN.

In addition to assessing the effect of preceding task on resting state, a novel aspect of this study is the classification analysis based on resting state functional connectivity. We were able to successfully identify participants as belonging to ASD or control groups in the current study with 77.78% accuracy. These results are similar to a recent study that used resting state to classify individuals with autism, with a classification accuracy of 79% [49]. Interestingly, when the same seed regions used in the deactivation results were used to classify groups based on functional connectivity, we found that the angular gyrus seed connectivity with the rest of the brain was able to identify participants’ group membership with 96.3% accuracy. Indeed, the angular gyrus was the only region that revealed group differences in regions, with individuals with ASD, compared to controls, showing underconnectivity between the left AG and visual and visual processing regions. In healthy controls, the left angular gyrus has been shown to have increased connectivity between visual processing regions, such as the fusiform gyrus, during resting state, and is suggestive of readying the visual system for processing phonological and semantic information of anticipated words [50]. In addition, the left angular gyrus has been shown to be involved in imagery and retrieval of visual information [51] and memory retrieval during rest [52]. It is possible that the difficulty individuals with autism have in modulating the deactivation of the default mode network, as we stated previously, may be making it difficult for them to anticipate and ready themselves mentally for an upcoming task.

Our results indicate that the pattern of abnormal DMN connectivity in ASD is specific enough to be able to distinguish it from the default connectivity network of the typical brain. Furthermore, this suggests that the default mode functional underconnectivity can emerge as a possible neural marker for autism. Indeed, a type of classification technique that assesses the DMN may be most useful for low-functioning individuals with ASD who are unable to participate in standard behavioral diagnostic tests. However, future research is needed to not only replicate our findings, but also to investigate the feasibility of biomarker classifications for diagnostic purposes.

It is important to note some of the limitations of this study. Firstly, while task-induced deactivation technique provides a unique avenue to determine the influence of task on resting state, it is slightly more vulnerable to noise and physiological artifacts when assessing functional connectivity. However, we attempted to correct this by following the Fair et al. [31] criteria for preprocessing our data. Nevertheless, it should be noted that our results were consistent with previous findings using different methods [5], [9]. Secondly, since we used fixation (rest) blocks in order to assess the DMN, there may have been carry-over effects from the cognitive tasks, reducing our power to detect group differences in functional deactivation. However, by using a ROI approach for our group analysis of the contrast rest vs. all cognitive tasks, we were able to increase our power to detect these differences. In addition, it should be noted that we used a very restricted age range of young adults in their late teens and early twenties, which may limit the generalizability of these results. In the future, in order to improve our understanding of this system in autism and to improve our classification accuracy, it would be helpful to track resting state differences throughout childhood when connectivity between brain regions is still developing. Lastly, we only assessed individuals with ASD and healthy controls so we are unable to speak as to whether this resting state profile in individuals with ASD can differentiate them from other developmental and neurological disorders (see [15] for review).

### Conclusions

In summary, we found that deactivation and connectivity of the DMN, suggestive of self-referential and mentalizing processes, were altered in individuals with ASD compared to typically developing control participants. Furthermore, these deficits appear to be mediated in part by (1) the limited ability of individuals with ASD to modulate the recruitment of DMN regions when engaging in a cognitive task that requires the individual to make social inferences about others, and (2) decreased connectivity of the dorsal MPFC, a key region in mentalizing, with the rest of the default mode connectivity network. In addition, we found that the deficits seen in the DMN in individuals with ASD can be used as a distinguishing feature, specifically in the angular gyrus, that can classify an individual as belonging in the ASD group. Indeed, this classification method may have clinical implications for low-functioning individuals with ASD who are unable to participate in standard behavioral testing. Overall, our findings suggest more in-depth examination of the DMN in autism to better understand not only the resting brain, but also its relationship to social cognition in autism.

## Supporting Information

Figure S1 Task activation maps for the ASD group for each of the three tasks at a p<0.001, FDR corrected threshold. For the contrast *self-referential language task vs. fixation* (labeled as **self-language**), the key regions activated included left dorsolateral prefrontal cortex (DLPFC), left inferior frontal gyrus (IFG), left superior frontal gyrus (SFG), left cuneus, and left inferior occipital gyrus (IOG). For the contrast *sentence language task vs. fixation* (labeled as **language**), the key regions of activation included bilateral DLPFC, left IFG, left SFG, left middle temporal gyrus (MTG), left superior temporal gyrus (STG), bilateral inferior parietal lobule (IPL), and left middle occipital gyrus (MOG). Lastly, for the *theory-of-mind task vs. fixation* (labeled as **theory of mind**), the key regions activated included right lingual gyrus, right IPL, bilateral IFG, and left DLPFC.
(TIF)

Figure S2 Task activation maps for the control group for each of the three tasks at a p<0.001, FDR corrected threshold. For the contrast *self-referential language task vs. fixation* (labeled as **self-language**), the key regions activated included left medial prefrontal cortex (MPFC), left DLPFC, left IFG, left middle temporal gyrus (MTG), bilateral cuneus, left IOG, and left MOG. For the contrast *sentence language task vs. fixation* (labeled as **language**), the key regions activated included left middle frontal gyrus (MFG), left SFG, left MTG, left precentral gyrus, bilateral cuneus, and bilateral lingual gyrus. Lastly, for the *theory-of-mind task vs. fixation* (labeled as **theory of mind**), the key regions activated included bilateral MFG, bilateral IFG, right IPL, right cuneus, left lingual gyrus, and left fusiform gyrus.
(TIF)

Figure S3 The most informative connections in ASD and control identification. Connections are illustrated on the center of mass of each AAL region. Red lines indicate positive correlation and blue lines indicate negative correlations. Line width indicates the correlation strength averaged across participants, ranging from −0.357 to 0.839 for the ASD group, and −0.367 to 0.794 for the control groups.
(TIF)

Figure S4 The most informative connections in ASD and control identification. Connections are illustrated on the center of mass of each AAL region. Red lines indicate autism and blue lines indicate control. Line width indicates the average weights assigned by the classifier, ranging from 0.002 to 0.061 for both groups.
(TIF)
