# Supplementary figures and images for: Differential Deactivation during Mentalizing and Classification of Autism Based on Default Mode Network Connectivity
Source: PLoS One. 2012 Nov 19;7(11):e50064. doi: 10.1371/journal.pone.0050064 (PMC3501481; doi:10.1371/journal.pone.0050064)

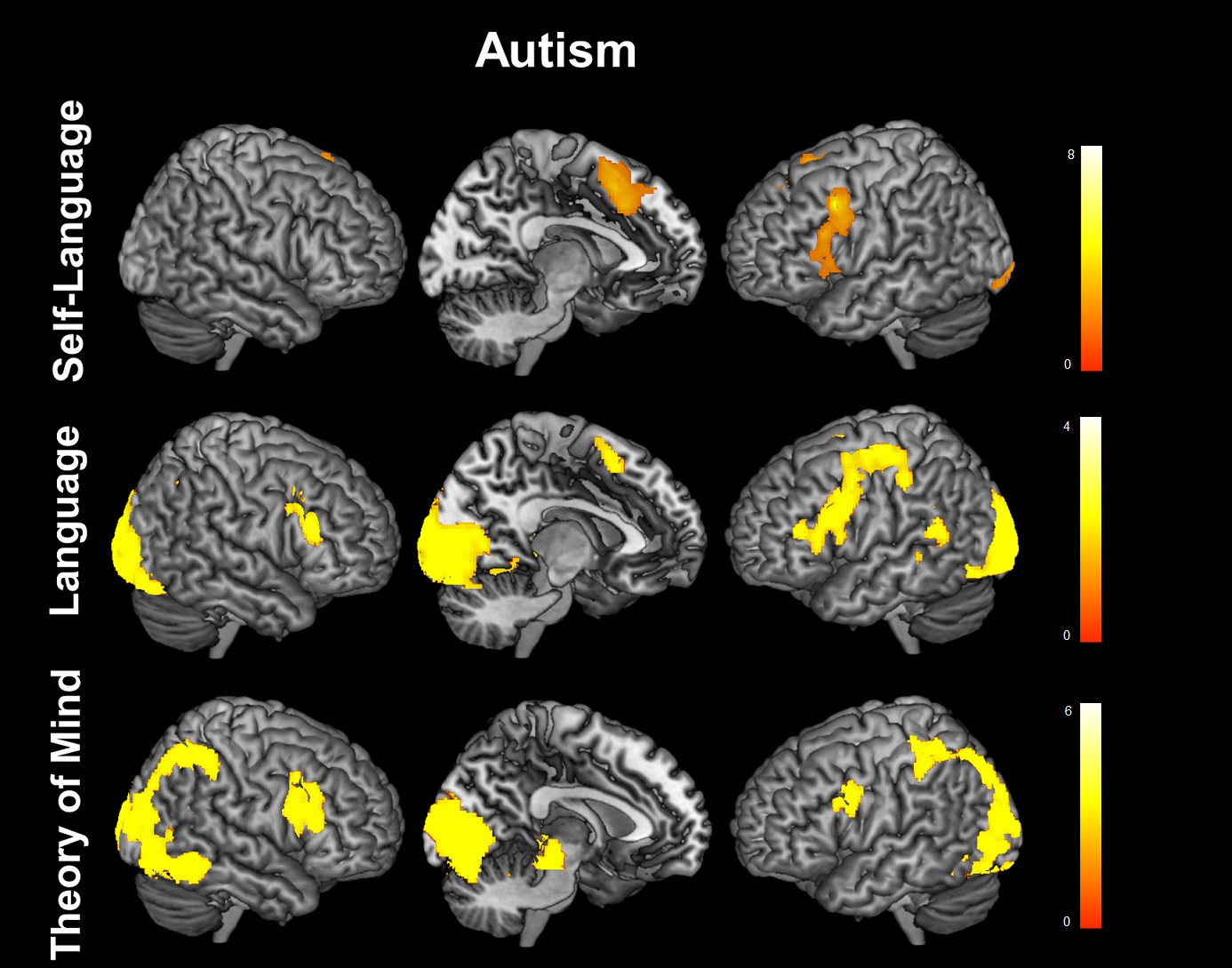

Supplement: Figure S1 — Task activation maps for the ASD group for each of the three tasks at a p<0.001, FDR corrected threshold. For the contrast self-referential language task vs. fixation (labeled as self-language), the key regions activated included left dorsolateral prefrontal cortex (DLPFC), left inferior frontal gyrus (IFG), left superior frontal gyrus (SFG), left cuneus, and left inferior occipital gyrus (IOG). For the contrast sentence language task vs. fixation (labeled as language), the key regions of activation included bilateral DLPFC, left IFG, left SFG, left middle temporal gyrus (MTG), left superior temporal gyrus (STG), bilateral inferior parietal lobule (IPL), and left middle occipital gyrus (MOG). Lastly, for the theory-of-mind task vs. fixation (labeled as theory of mind), the key regions activated included right lingual gyrus, right IPL, bilateral IFG, and left DLPFC. (TIF) [file pone.0050064.s001.tif]

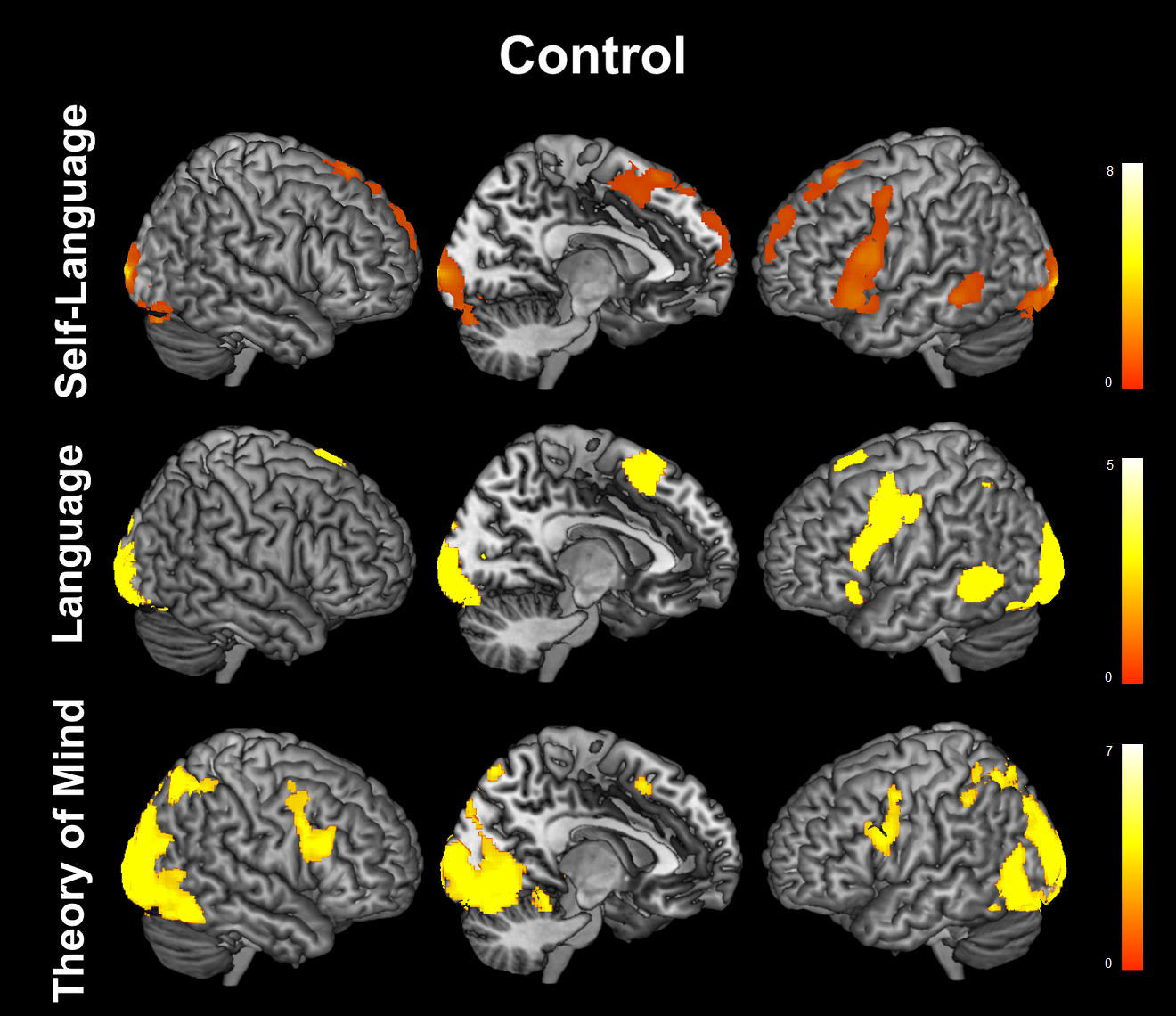

Supplement: Figure S2 — Task activation maps for the control group for each of the three tasks at a p<0.001, FDR corrected threshold. For the contrast self-referential language task vs. fixation (labeled as self-language), the key regions activated included left medial prefrontal cortex (MPFC), left DLPFC, left IFG, left middle temporal gyrus (MTG), bilateral cuneus, left IOG, and left MOG. For the contrast sentence language task vs. fixation (labeled as language), the key regions activated included left middle frontal gyrus (MFG), left SFG, left MTG, left precentral gyrus, bilateral cuneus, and bilateral lingual gyrus. Lastly, for the theory-of-mind task vs. fixation (labeled as theory of mind), the key regions activated included bilateral MFG, bilateral IFG, right IPL, right cuneus, left lingual gyrus, and left fusiform gyrus. (TIF) [file pone.0050064.s002.tif]

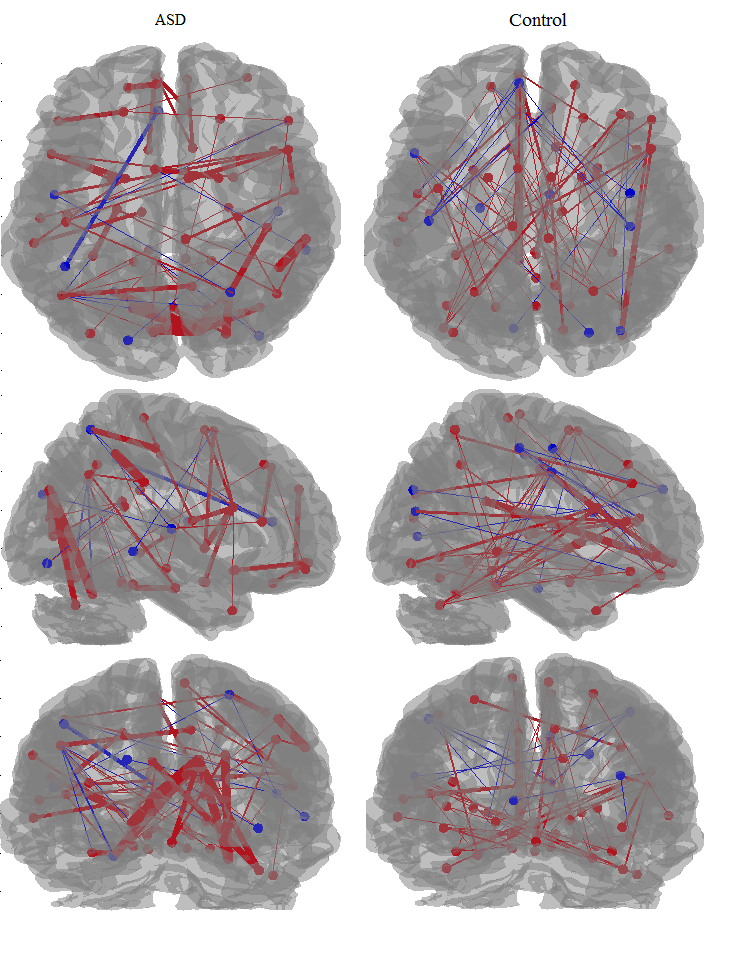

Supplement: Figure S3 — The most informative connections in ASD and control identification. Connections are illustrated on the center of mass of each AAL region. Red lines indicate positive correlation and blue lines indicate negative correlations. Line width indicates the correlation strength averaged across participants, ranging from −0.357 to 0.839 for the ASD group, and −0.367 to 0.794 for the control groups. (TIF) [file pone.0050064.s003.tif]

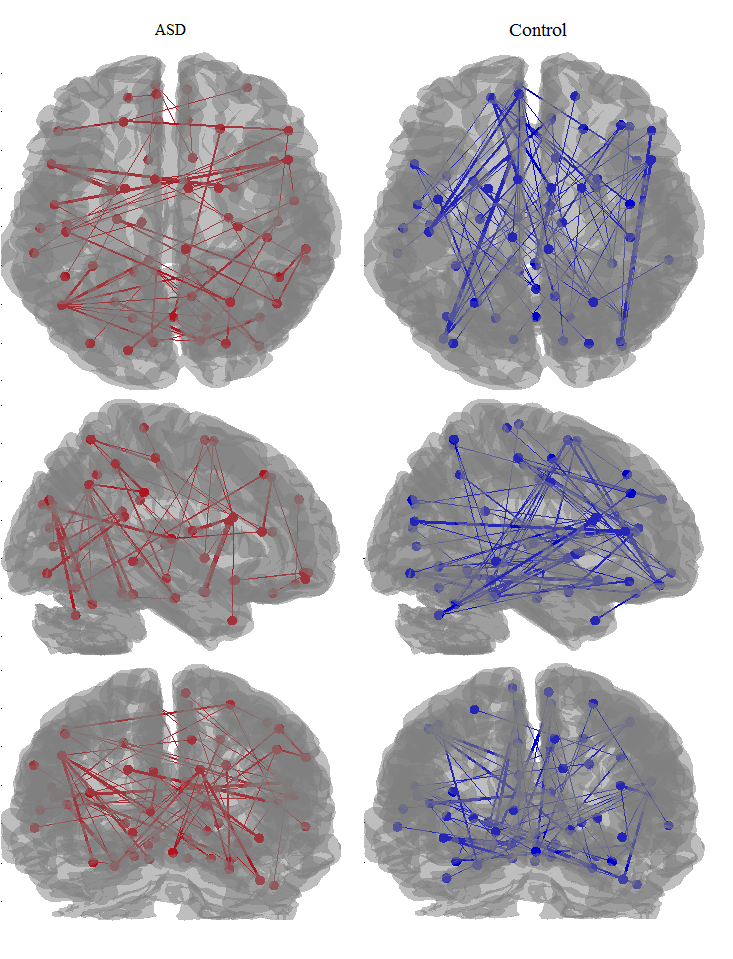

Supplement: Figure S4 — The most informative connections in ASD and control identification. Connections are illustrated on the center of mass of each AAL region. Red lines indicate autism and blue lines indicate control. Line width indicates the average weights assigned by the classifier, ranging from 0.002 to 0.061 for both groups. (TIF) [file pone.0050064.s004.tif]
